# Sex‐specific genetic regulation of proteomics in cerebrospinal fluid uncovers genetic causes for sex differences in neurodegeneration

**DOI:** 10.1002/alz.089696

**Published:** 2025-01-03

**Authors:** Yun Ju Sung, Lihua Wang, Daniel Western, Jigyasha Timsina, Priyanka Gorijala, Agustin Ruiz, Pau Pastor, Han Chen, Carlos Cruchaga

**Affiliations:** ^1^ NeuroGenomics and Informatics Center, Washington University School of Medicine, St. Louis, MO USA; ^2^ NeuroGenomics & Informatics Center, St. Louis, MO USA; ^3^ NeuroGenomics & Informatics Center, Washington University School of Medicine, St. Louis, MO USA; ^4^ Washington University School of Medicine, Saint Louis, MO USA; ^5^ Research Center and Memory Clinic, Fundació ACE Institut Català de Neurociències Aplicades ‐ Universitat Internacional de Catalunya (UIC), Barcelona Spain; ^6^ Memory Disorders Unit, Department of Neurology, Hospital Universitari Mutua de Terrassa, Terrassa, Barcelona, Terrassa Spain; ^7^ Human Genetics Center, Department of Epidemiology, Human Genetics and Environmental Sciences, School of Public Health, The University of Texas Health Science Center at Houston, Houston, TX USA; ^8^ Neurogenomics & Informatics Center, St. Louis, MO USA

## Abstract

**Background:**

Clear sex differences exist in AD and PD. Several studies examined genetic regulations for AD phenotypes and gene expression data in a sex‐specific manner, identifying some differences between males and females. In contrasts, although proteins are final effectors of most physiological pathways and important drug targets, sex‐specific regulations for proteins remain vastly understudied. This study therefore aims to investigate sex‐specific genetic regulation of proteomics in neurologically relevant cerebrospinal fluid (CSF).

**Method:**

We recently identified 3,885 protein quantitative loci (pQTL) for 1,883 proteins, many of which were unique to CSF, demonstrating genetic regulation specific to the CSF proteome. Here, we performed a sex‐stratified pQTL (in 1,640 males and 1,713 females, separately) with >7000 proteins (SomaScan7K) on ∼13 million TOPMED imputed autosomal variants. pQTL considered significant at P<5 × 10^‐8^ (in cis) and P<3.45 × 10^‐11^ (in trans) in either sex. We subsequently examined shared genetic regulation using colocalization with neurodegeneration and psychiatric diseases.

**Result:**

We identified 3,179 significant pQTLs (1,210 cis and 1,969 trans) in either sex in 726 genetic loci for 1,794 proteins. While 69.4% of pQTLs had similar regulation in both sexes, 21.1% (173 female‐specific; 220 male‐specific) were significant only in one sex. For example, pQTL for IST1 at rs71709684 (MAF = 0.047) on chr16 was female‐specific, significant only in females (β = 0.43, P = 4.40 × 10^‐8^ in females vs β = ‐0.02, P = 7.89 × 10^‐1^ in males). In addition, 8.5% (47 female‐biased; 97 male‐biased) were significant in both, but with different magnitudes. The PD locus *LRRK2* on chr12 regulated several proteins (GRN, GPNMB, ITGB2), enriched in microglia, in males only. As the most pleiotropic region, the *APOE* region on chr19, the strongest AD locus, sex‐specifically regulated multiple proteins relevant to AD (PPP3R1, YWHAZ, PPP3CA, amyloidosis‐related NRXN2, VSNL1, and dementia‐related TIMM8A), enriched in neurons. The female‐specific pQTL of PLOD3 on chr17 colocalized with alcoholism, testosterone levels, and sex hormone‐binding globulin (SHBG). The male‐specific pQTL of NRXN2 on chr19 colocalized with AD, along with SHBG and telomere length.

**Conclusion:**

By performing sex‐stratified pQTL, we uncovered distinctive sex‐specific genetic regulation of CSF proteome. Our findings complement sex‐aware eQTL from GTEx and provide insights on dissecting the underlying biological causes and mechanisms contributing to sex differences in neurodegeneration.

